# Smooth Muscle in Cardiac Chambers is Common in Turtles and Extensive in the Emydid Turtle, *Trachemys scripta*

**DOI:** 10.1002/ar.24257

**Published:** 2019-10-10

**Authors:** William Joyce, Dane A. Crossley, Tobias Wang, Bjarke Jensen

**Affiliations:** ^1^ Zoophysiology, Department of Bioscience Aarhus University, 8000 Aarhus C Denmark; ^2^ Department of Medical Biology, Amsterdam UMC University of Amsterdam, Amsterdam 1105AZ the Netherlands; ^3^ Aarhus Institute of Advanced Studies Aarhus University Aarhus Denmark; ^4^ Department of Medical Biology University Medical Center Amsterdam Amsterdam The Netherlands

**Keywords:** cardiac function, diving physiology, stroke volume, heart anatomy

## Abstract

A prominent layer of smooth muscle lining the luminal side of the atria of freshwater turtles (Emydidae) was described more than a century ago. We recently demonstrated that this smooth muscle provides a previously unrecognized mechanism to change cardiac output in the emydid red‐eared slider (*Trachemys scripta*) that possibly contributes to their tremendous diving capacity. The purpose of the present immunohistochemical study was firstly to screen major groups of vertebrates for the presence of cardiac smooth muscle. Secondly, we investigated the phylogenetic distribution of cardiac smooth muscle within the turtle order (Testudines), including terrestrial and aquatic species. Atrial smooth muscle was not detected in a range of vertebrates, including *Xenopus laevis*, *Alligator mississippiensis*, and *Caiman crocodilus*, all of which have pronounced diving capacities. However, we confirmed earlier reports that traces of smooth muscle are found in human atrial tissue. Only within the turtles (eight species) was there substantial amounts of nonvascular smooth muscle in the heart. This amount was greatest in the atria, while the amount in proportion to cardiac muscle was greater in the sinus venosus than in other chambers. *T. scripta* had more smooth muscle in the sinus venosus and atria than the other turtles. In some specimens, there was some smooth muscle in the ventricle and the pulmonary vein. Our study demonstrates that cardiac smooth muscle likely appeared early in turtle evolution and has become extensive within the Emydidae family, possibly in association with diving. Across other tetrapod clades, cardiac smooth muscle might not associate with diving. Anat Rec, 303:1327–1336, 2020. © 2019 The Authors. *The Anatomical Record* published by Wiley Periodicals, Inc. on behalf of American Association for Anatomy.

In the late 19th century, slow wave contractions (the so‐called tonus waves) were observed in isolated atrial preparations from European pond turtles (*Emys orbicularis*) (Fano, 1887; Fano and Fayod, 1888; Bottazzi, 1897). The tonus waves were soon attributed to the conspicuous amounts of smooth muscle in the atria (Rosenzweig, 1903; Bottazzi, 1906), but despite a few decades of relatively intense research into its pharmacological properties (Fano, 1887; Fano and Fayod, 1888; Bottazzi and Grünbaum, 1899; Gault, 1917; Gruber and Markel, 1918a, 1918b; Gruber, 1920a, 1920b, 1921, 1927; Sollmann and Rossides, 1927; Gruber, 1934; Dimond, 1959), the scientific interest in the atrial smooth muscle waned after the 1920s. As part of other studies, we also observed the tonus waves (Galli *et al*., 2006; Joyce *et al*., 2014), and with a revived curiosity into their functional role, we recently demonstrated that the atrial smooth muscle may provide a powerful means to regulate ventricular filling and hence cardiac stroke volume (Joyce *et al*., 2019). We predicted that atrial smooth muscle enables fine‐tuning of ventricular filling and thus stroke volume during the characteristic rapid transitions from slow heart rates in apnea to the tachycardia associated with intermittent lung ventilation (Wang and Hicks, 1996; Joyce *et al*., 2018). Our previous experiments (Joyce *et al*., 2018) were designed with the atria in mind and we could not assess the presence and impact of smooth muscle in the sinus venosus or veins.

The European‐based discoverers of the atrial smooth muscle universally employed the Emydid turtle *E. orbicularis* (formerly *Emys europaea*) (e.g., Fano, 1887; Bottazzi and Grünbaum, 1899; Fano and Bodano, 1900; Rosenzweig, 1903; Oinuma, 1910), but were soon followed by North American studies on a wealth of other turtle species in the Emydid family, including *Trachemys scripta* (Laurens, 1913; Gruber and Markel, 1918a, 1918b; Pereira, 1924; Sollmann and Rossides, 1927; Robb, 1952; Dimond, 1959). There are additional vague descriptions of tonus waves in atria from *Chelydra serpentina* (snapping turtles; Chelydridae); very little detail is given by Pereira (Pereira, 1924), where data are indiscriminately combined with findings in Emydid turtles, while Blinks and Koch‐Weser (1963) cite their own unpublished observations about this species. Gaskell (1900) did not observe tonus waves in the atria of the land tortoise (Testudinidae), *Testudo graeca*. In a more recent conference abstract, Gannon *et al*. (2003)) did not locate atrial smooth muscle in a side‐necked turtle (Pleurodira), *Emydura macquarii*, although it has been detected in the sinus venosus, the chamber upstream of the right atrium in other reptile hearts (Jensen *et al*., 2014, 2017). If atrial smooth muscle is functionally related to cardiovascular regulation during diving, we predict it would be prevalent in aquatic species that exhibit large changes in heart rate and cardiac output during ventilation, but absent or less conspicuous in terrestrial species where the cardiorespiratory interactions are smaller (Glass *et al*., 1978; Taylor and Wang, 2009).

Bottazzi (1897) observed tonus waves in atrial preparations of anuran amphibians (frogs and toad), but atrial smooth muscle has not been detected histologically (Laurens, 1913; Blinks and Koch‐Weser, 1963). Also, there are numerous reports of endocardial smooth muscle in human (Nagayo, 1909; Blinks and Koch‐Weser, 1963; Park *et al*., 2013; Okada *et al*., 2015) and sheep (Terasaki *et al*., 1993). Possibly, this reflects contributions of the Isl1‐positive second heart field to the venous pole of the heart and the common origin of smooth muscle and cardiac muscle in mesodermal progenitors that express *Isl1* (Douglas *et al*., 2006, 2009; Moretti *et al*., 2006).

The primary aim of this study was to unravel the evolutionary history of atrial smooth muscle in Testudines. To test the hypothesis that the smooth muscle may be linked to diving capacity, we predicted that atrial smooth muscle would be absent in terrestrial tortoises, but more developed in aquatic species. We also took the opportunity to describe smooth muscle in other parts of the heart, including the sinus venosus (Carmona et al., 2018), pulmonary veins, and ventricle. We finally considered the possible broader distribution of atrial smooth muscle in other vertebrates, including amphibians and mammals.

## MATERIALS AND METHODS

The majority of the turtle species (*Pelomedusa subrufa* [*n* = 3; 20–35 g], *Chelodina mccordi* [*n* = 3; 14–15 g], *Pelodiscus sinensis* [*n* = 2; 5 g]*, Cyclanorbis senegalensis* [*n* = 2; 0.2–0.45 kg], *Testudo hermanii* [*n* = 3; 25–27 g], *Chelonoidis carbonaria* [*n* = 3; 2.4–4.8 kg], and *T. scripta* [*n* = 10; 0.3–1.7 kg], a skink, *Cyclodomorphus gerrardii* [*n* = 1; 0.44 kg], a spectacled caiman, *Caiman crocodilus* [*n* = 1; 4 kg], African clawed frogs, *Xenopus laevis* [*n* = 2; 50 g], cane toads, *Rhinella marinus* [*n* = 2; 100–200 g]) and Longnose gar (*Lepisosteus osseus* [n = 1])were obtained from commercial sources or donated from private collections and maintained at the Aarhus University (Aarhus, Denmark). *C. serpentina* (*n* = 3; 30–35 g) and *Alligator mississippiensis* (*n* = 1; 1 kg) hearts were obtained from animals maintained at the University of North Texas (Denton, Texas). Mouse (*Mus musculus* [*n* = 1]), and a bird, the lesser redpoll (*Acanthis cabaret* [N = 1]), sections were obtained from archived samples (body mass unknown) at the Amsterdam University Medical Center (UMC) (Amsterdam, the Netherlands). One caecilian (*Idiocranium* sp.) section was taken from unpublished data associated with an earlier study (de Bakker *et al*., 2015). Healthy human (*Homo sapiens*) cardiac samples were provided from the Department of Pathology, Amsterdam UMC, AMC (Amsterdam, the Netherlands).

For the hearts used for immunohistochemistry, the animals were euthanized with an overdose of pentobarbital (200 mg kg^−1^) before the brain was destroyed. All experiments were performed in accordance with local animal care regulations.

### Immunohistochemistry

Hearts were fixed for 24 hr in paraformaldehyde (4% in phosphate‐buffered saline [PBS] ) and stored in 70% ethanol. The hearts were then embedded in paraffin (Paraplast, Sigma P3558) and cut into 10‐μm transverse or coronal sections. A standard immunohistochemistry protocol was followed as described elsewhere (Jensen *et al*., 2016; 2017), where we demonstrated specific detection of cardiac muscle and smooth muscle in anole lizards, the Ball python, and the American alligator. Briefly, cardiac muscle was detected with rabbit polyclonal antibody to cardiac Troponin I (cTnI: 06/02‐IV‐4T21/2, HyTest Ltd, dilution 1:600) which was detected with a secondary donkey anti‐rabbit antibody conjugated to the fluorophore Alexa 647 nm (Mol Prob A31573, dilution 1:250). Smooth muscle was detected with mouse monoclonal antibody to smooth muscle actin (SMA, Sigma A2547, 1:600) which was detected with a secondary donkey anti‐mouse antibody conjugated to the fluorophore Alexa 555 nm (Invitrogen A31570, 1:250). Images were acquired with a Leica DM6000 microscope under the control of LAS X software (Leica Microsystems, Wetzlar, Germany).

### Statistical Analyses

The number of pixels containing myocardium (red) and smooth muscle (green) in composite images were determined by splitting the red and green colors using the “Color Threshold” function of ImageJ (NIH, Bethesda, MD, Version 1.51k), and then measuring the area on the split colors allowing us to calculate relative smooth muscle area as a percentage of the total muscle area (smooth and cardiac muscle). To maintain standardization, only transverse images of the atria were used for this quantification, thus the final sample sizes in this analysis were as follows: *P. subrufa* (*n* = 2), *C. mccordi* (*n* = 2), *P. sinensis* (*n* = 1), *C. senegalensis* (*n* = 2), *C. serpentina* (*n* = 2), *T. hermanii* (*n* = 2), *C. carbonaria* (*n* = 3) and *T. scripta* (*n* = 6). For each heart, the % area of smooth muscle was averaged from three or four equidistant sections from across the atria, although in some *C. mccordi* (*n* = 1), *P. sinensis* (*n* = 1), and *C. carbonaria* (*n* = 2) only two representative sections could be used. Due to the low animal sample sizes available for most species, no statistical comparisons were made between species. A linear regression was performed to investigate the relationship between body mass and atrial smooth muscle coverage in *T. scripta*. To investigate whether there were chamber differences in the proportion of the detected SMA relative to all detected SMA and cTnI, we used the Plugin function “RGB Measure” of ImageJ after having delineated the sinus venosus, atria, or ventricle with the Freehand selections tool (epicardial vessels were excluded from this analysis to the extent it was possible). The output value (mean) is a composite measure of the number of pixels that contain each color and the color intensity. We only used images that contained both sinus venosus and atria (N = 73). In 25 of the 73 images, there was also ventricular tissue. Differences between the sinus venosus and atria were tested with paired *T*‐test. We used the Pearson correlation test for significant relation between the proportion of SMA in the sinus venosus as compared to the atria and ventricle. Statistical analyses were performed in SPSS (IBM SPSS Statistics version 24) or GraphPad Prism (Version 8.0). Data are presented as means ± SD.

## RESULTS

### Cardiac Smooth Muscle in Vertebrates

**Figure 1:**
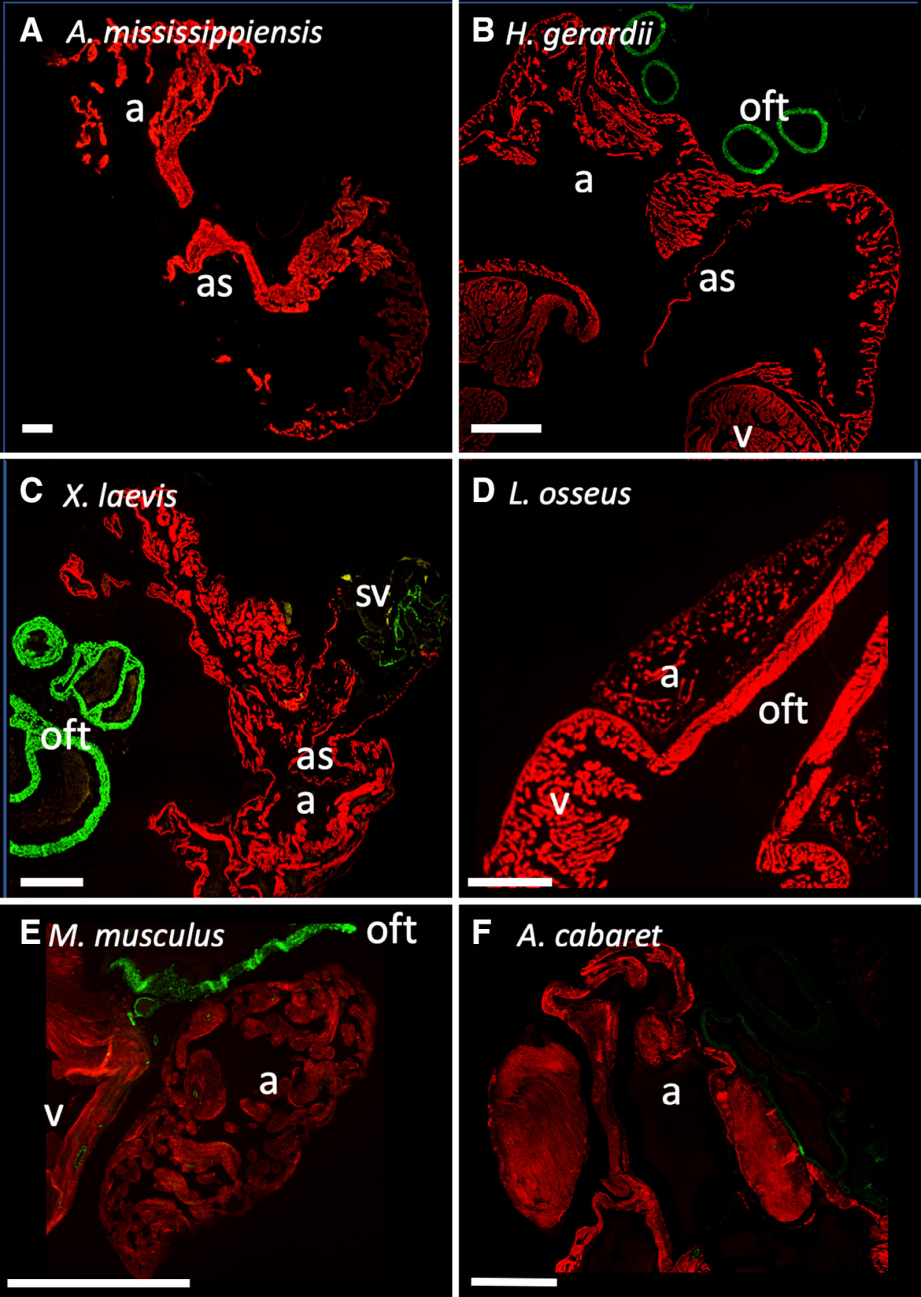
Luminal atrial smooth muscle was not detected in most vertebrates. Red represents cTnI and green represents SMA, as detected by fluorescent immunohistochemistry. (**A**) American alligator, (**B**) pink‐tongued skink, (**C**) African clawed frog, (**D**) longnose gar, (**E**) mouse, and (**F**) lesser redpoll bird (all detected SMA in B, E, F was within arterial walls). Scale bars are 1 mm. a, atrium; as, atrial septum; pv, pulmonary vein; sv, sinus venosus; v, ventricle; oft, outflow tract.

**Figure 2:**
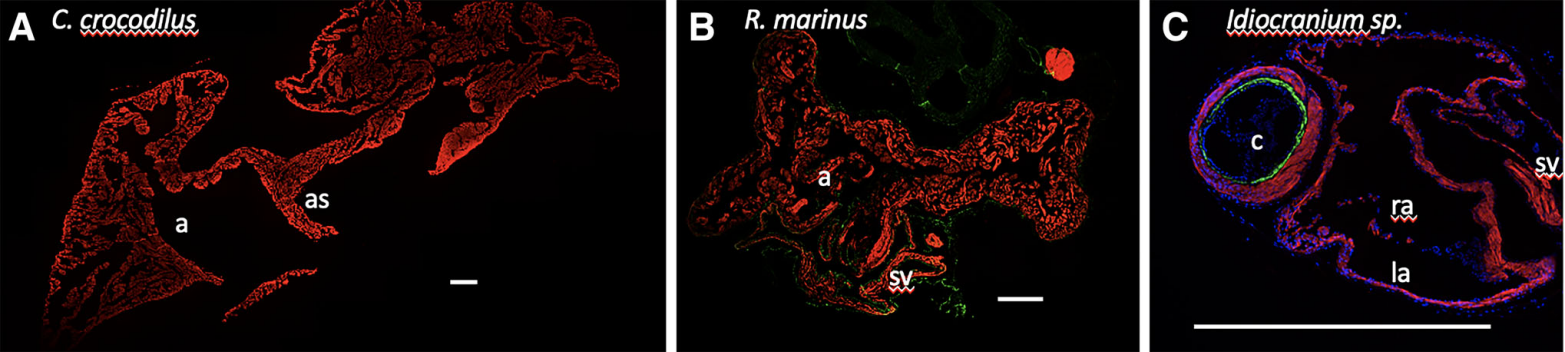
Luminal smooth muscle was not detected in atrium from spectacled caiman (**A**), cane toad (**B**), or caecilian (**C**). Red represents cTnI and green represents SMA, as detected by fluorescent immunohistochemistry. Scale bars are 1 mm. (r/l)a, (right/left) atrium; as, atrial septum; sv, sinus venosus; c, conus.

**Figure 3:**
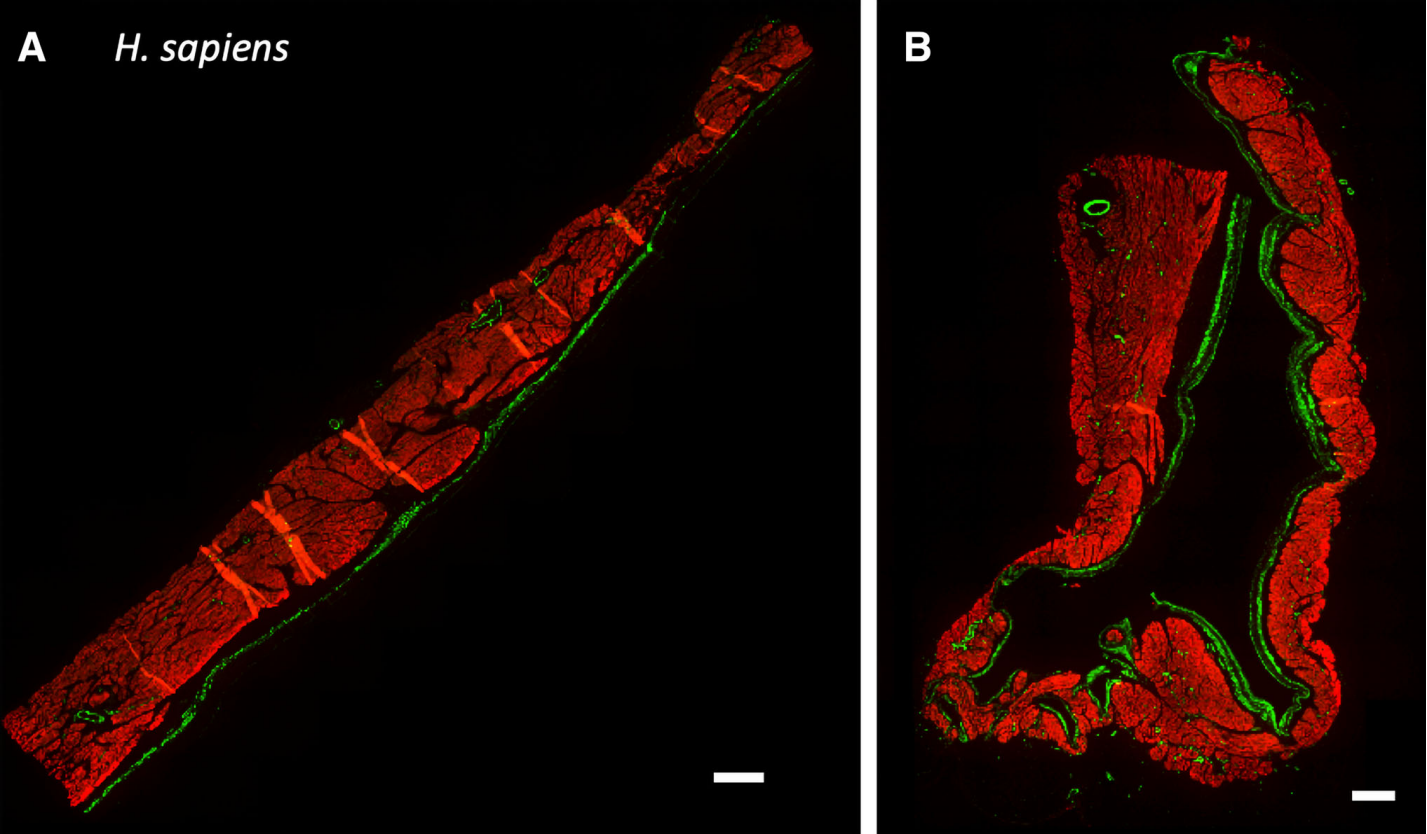
Smooth muscle in human atrial wall. Red represents cardiac cTnI and green represents SMA, as detected by fluorescent immunohistochemistry. (**A**) left anterior atrium and (**B**) left atrial appendage. Scale bars are 1 mm.

To resolve the evolutionary history of smooth muscle in the heart, we selected a range of vertebrate species and performed fluorescent immunohistochemistry against SMA and cardiac muscle in the sinus venosus, atria, and ventricle. Smooth muscle was readily detected in the large arteries connected to the ventricle and coronary arteries of all investigated species. Luminal smooth muscle was not detected in cardiac chambers from American alligator (*Alligators mississipinesis*) (Fig. 1A), spectacled caiman (*C. crocodilus*) (Fig. 2A), pink‐tongued skink (*C. gerrardii*) (Fig. 1B), longnose gar (*L. osseus*) (Fig. 1D), mouse (*M. musculus*) (Fig. 1E), or lesser red poll bird (*A. cabaret*) (Fig. 1F). Only in the African clawed frog (*X. laevis*) (Fig. 1C) and cane toad (*R. marinus*) (Fig. 2B) did we detect a small amount of smooth muscle in the sinus venosus, although none was observed in another amphibian, the caecilian (*Idiocranium* sp.) (Fig. 2C). We confirmed earlier reports (Nagayo, 1909; Park *et al*., 2013) that atrial smooth muscle could be detected in human atrium (Fig. 3), but most vertebrates appear to have very little, if any, smooth muscle in contact with chamber lumens.

### Cardiac Smooth Muscle in Turtles

**Figure 4:**
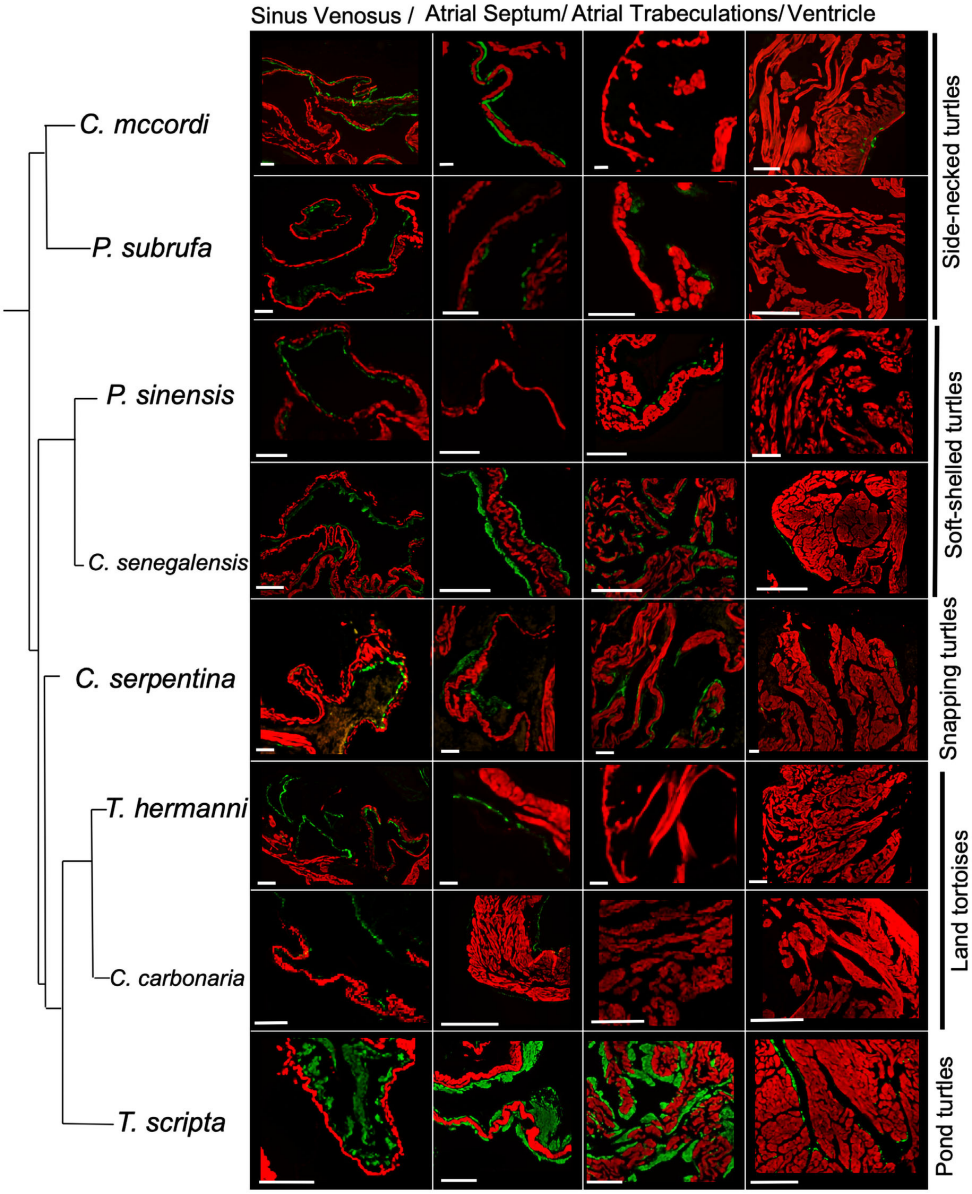
The phylogenetic distribution of smooth muscle in different regions of the heart in eight turtle species. Red represents cTnI and green represents SMA, as detected by fluorescent immunohistochemistry. Scale bars are 100 μm for all species, except for *C. senegalensis* and *T. scripta* (500 μm), and *C. carbonaria* (1 mm). Phylogeny based on Crawford *et al*. (2015).

**Figure 5:**
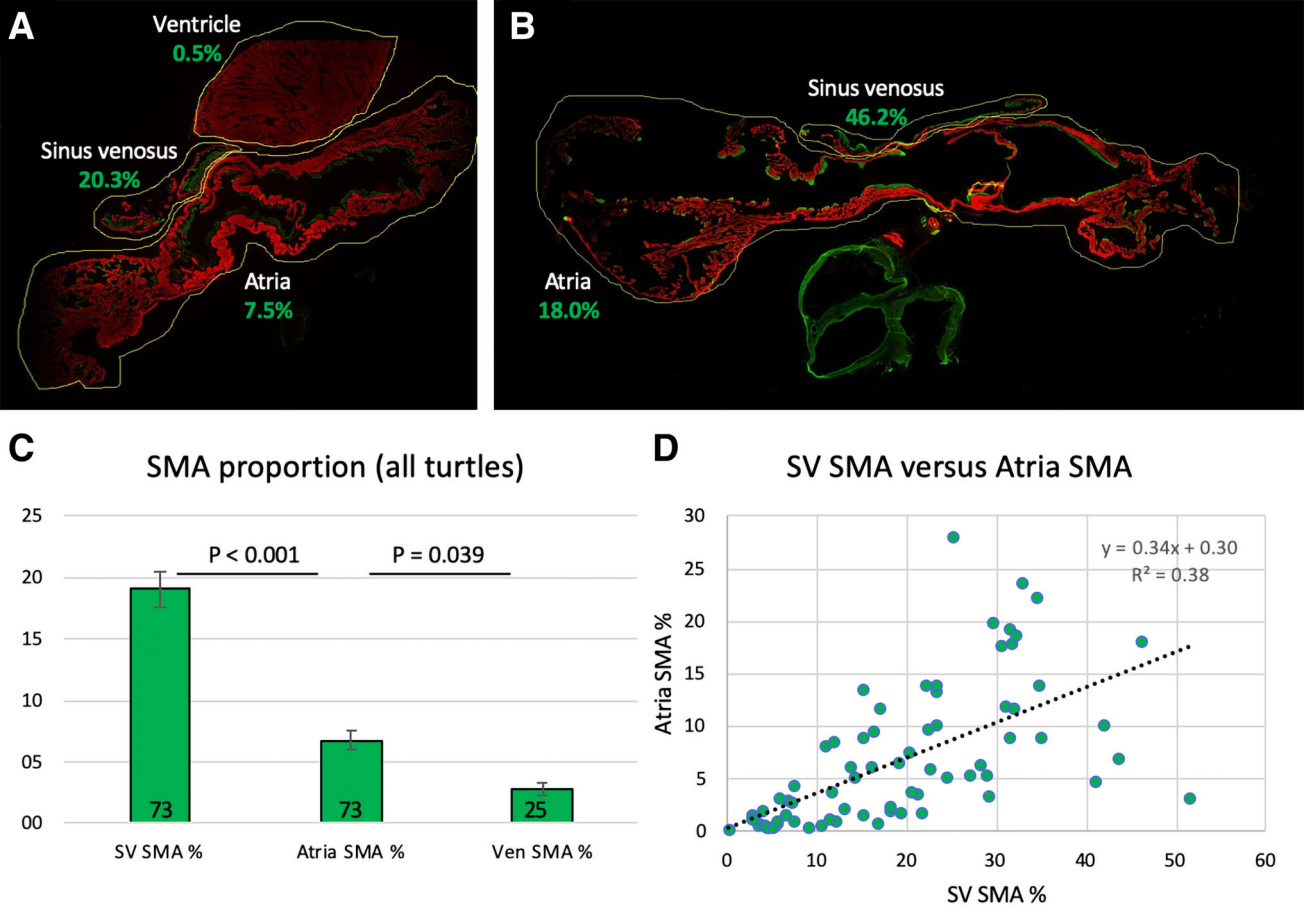
Assessment across turtles of the proportion of signal from SMA (green) out the total signal from SMA and cTnI (red), measured by the ImageJ Plugin function “RGB measure.” The yellow lines indicate the region of interest within which the measurements were made. Two specimens of the Pond slider with intermediate **(A)** and high **(B)** proportions (numbers in green) of SMA in the sinus venosus and atria. (**C**) Across turtles, the sinus venosus was proportionally richer in SMA than the atria, which in turn were richer in SMA than the ventricle (*P*‐values of paired two‐tailed *T*‐tests, numbers in columns are the number of assessed sections). (**D**) The proportion of SMA in the sinus venosus was significantly positively related to the proportion of SMA in the atria (Pearson correlation, *P* < 0.001).

**Figure 6:**
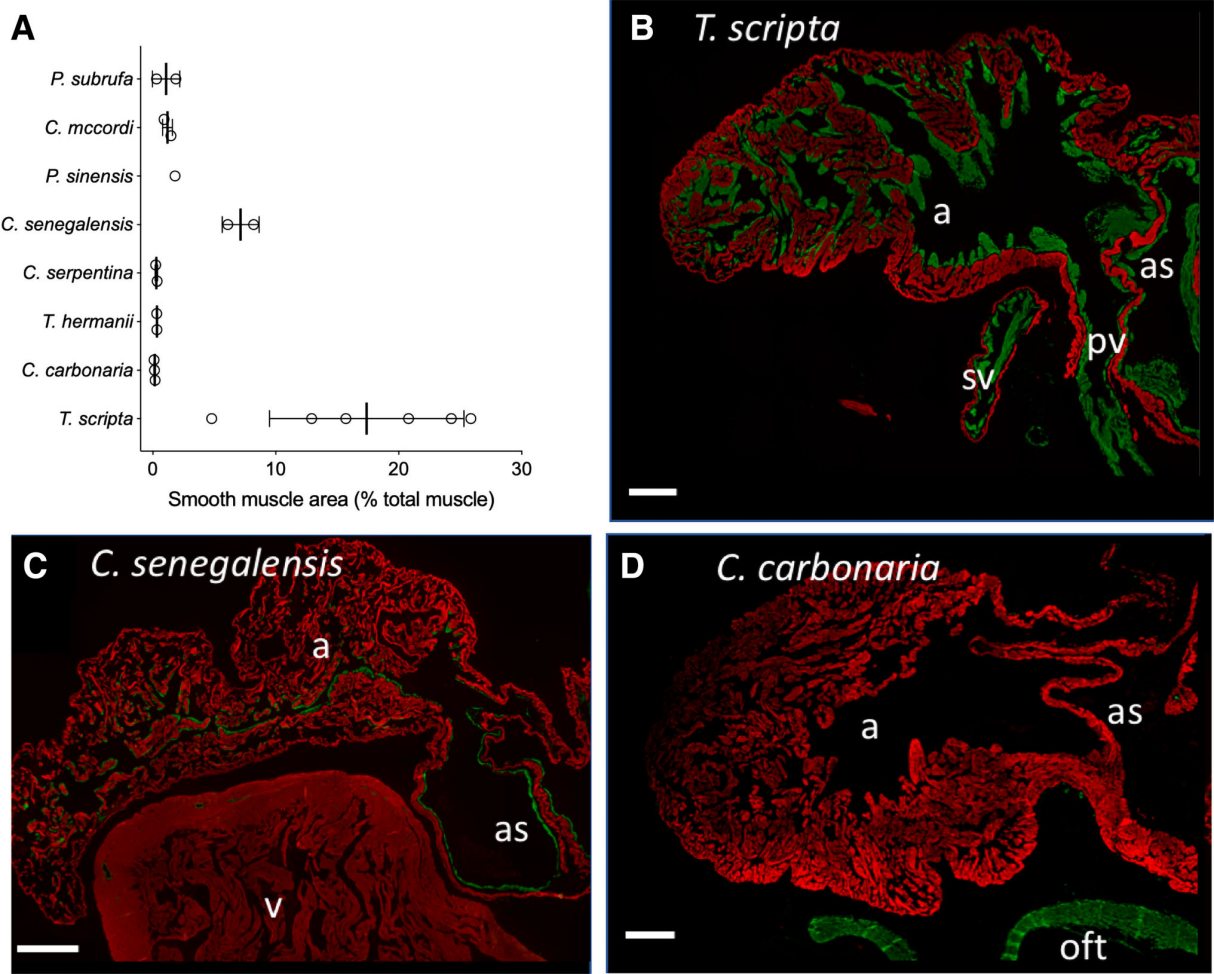
The interspecific variation in atrial smooth muscle in turtles. (**A**) Mean area of smooth muscle as a percentage of total muscle (smooth + cardiac muscle) area in eight species. Values are means ± SD. (**B–D**) Red represents cardiac Troponin I and green represents smooth muscle actin, as detected by fluorescent immunohistochemistry. (B) A prominent case of atria smooth muscle in *T. scripta*. (C) Intermediate levels of smooth muscle seen in *C. senegalensis*. (D) Near absence of atrial smooth muscle in *C. carbonaria*. For B–D, scale bars are 1 mm. a, atrium; as, atrial septum; pv, pulmonary vein; sv, sinus venosus; v, ventricle; oft, outflow tract.

All eight turtle species exhibited both smooth and cardiac muscle in the sinus venosus (Fig. 4). In proportion to the total amount of smooth and cardiac muscle, the sinus venosus had significantly more smooth muscle than the atria, which in turn had significantly more smooth muscle than the ventricle (Fig. 5) (concerning the ventricle, in some specimens much of the SMA signal came from the walls of coronary arteries). The proportion of smooth muscle in the atria was positively related to the proportion of smooth muscle in the sinus venosus (Fig. 5), but the proportion of smooth muscle of the ventricle was not (Pearson correlation, *P* = 0.271). In *T. scripta* there was a great amount of smooth muscle in the sinus venosus and atria (Fig. 4) that represented 17.4 ± 7.9% (mean ± SD) of total muscle area (Fig. 6A). Smooth muscle was observed equally in the left and right atria, and appeared homogeneously within each atrium. Smooth muscle was also relatively prevalent (7.1 ± 1.5% total muscle area) in *C. senegalensis*, a distantly related soft‐shelled turtle (Fig. 6A,C). In the other species, smooth muscle on atrial trabeculae was sparser, although positive identifications were made in all species except land tortoises (*T. hermanii* and *C. carbonaria* Fig. 6D) and one of the side‐necked turtles, *C. mccordi* (Fig. 4). Notwithstanding, smooth muscle was identified on the atrial septum in all species except *P. sinensis*, in which a limited sample size may have precluded finding it, although it was minimal in *P. subrufa* and *C. carbonaria* (Fig. 4). In all species, except for *T. scripta* and *C. senegalensis*, the total contribution of smooth muscle averaged less than 2% total muscle area in the atria (including atrial septum) (Fig. 6A). In *T. scripta*, smooth muscle was also observed on ventricular trabeculae (Fig. 4), although this was clearly very much sparser than in the atria so was not the main focus of our study. Traces of smooth muscle were also found in the *C. senegalensis* and *C. serpentina* ventricle.

**Figure 7:**
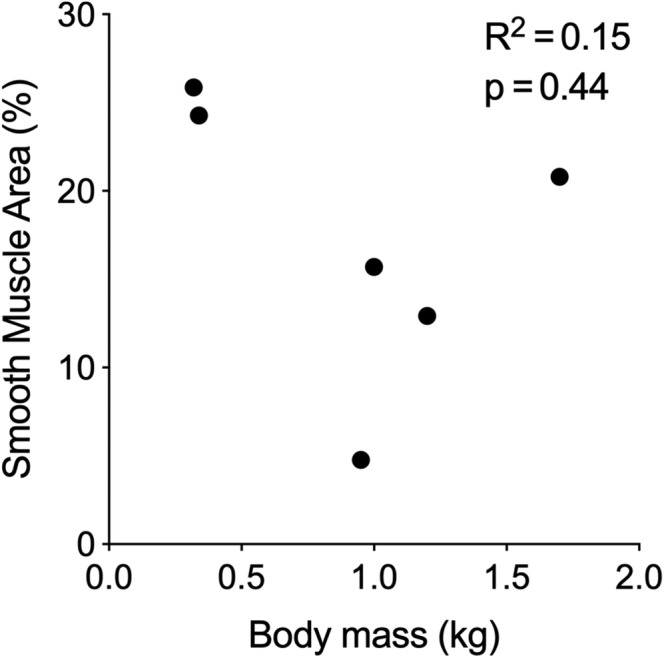
There was no significant relationship between atrial smooth muscle (% area of total muscle) and body mass in *Trachemys scripta* (linear regression).

Given the body mass range we encountered within and between species, in *T. scripta* we established that there was no relationship between body mass and atrial smooth muscle area (R^2^ = 0.15, *P* = 0.44) (Fig. 7). Also, in *T. scripta* there was no relation between the amount of SMA in the sinus venosus and the atria (in proportion to the total amount of smooth and cardiac muscle, R^2^ = 0.02). However, the proportional amount of SMA was greater in *T. scripta* compared to the other turtles in the sinus venosus (unpaired two‐tailed *T*‐test, unequal variance assumed, *P* < 0.001) and in the atria (unpaired two‐tailed *T*‐test, unequal variance assumed, *P* < 0.001) but not in the ventricle (unpaired two‐tailed *T*‐test, unequal variance assumed, *P* = 0.939).

## DISCUSSION

The cardiac output of *T. scripta* is dramatically reduced when the smooth muscle of the atria is induced to contract (Joyce *et al*., 2019) and we demonstrate here an extensive layer of smooth muscle in atria of this species. We also reveal large amounts of smooth muscle in the sinus venosus, the cardiac chamber between the systemic veins and the right atrium (Jensen *et al*., 2014, 2017; Icardo, 2017), and we consider it likely that contraction of the smooth muscle of both chambers could reduce cardiac filling by impeding venous return. Cardiac filling may also be limited by reduced compliance of the chamber walls. From a functional point of view, there are least two similar adaptations to altering venous return among vertebrates. Diving mammals have a vena caval sphincter that may impede venous return during diving bradycardia (Harrison and Tomlinson, 1956; Elsner *et al*., 1971; Blix, 2018; Lillie *et al*., 2018) and some terrestrial snakes have a “corkscrew” caval vein that may facilitate venous return during gravitational challenges such as during climbing (Lillywhite, 1987; Conklin *et al*., 2009). In consistent with earlier descriptions (Shaner, 1923; Robb, 1952), smooth muscle was sparse but consistently identified in the ventricle of *T. scripta* (and minute amounts were found in *C. senegalensis and C. serpentina*).

### Smooth Muscle in the Atria of Turtles

Our results suggest that (nonvessel) cardiac smooth muscle appeared early in turtle evolution, possibly in the sinus venosus before other chambers, as it was observed, at least in small amounts, in representatives across the turtle phylogeny, including side‐necked turtles (Pleurodira) that diverged from other turtles over 150 million years ago (Crawford *et al*., 2015; Shaffer *et al*., 2017). It was not, however, observed in other reptiles, including crocodilians or birds, which as archosaurs represent the closest living relatives to turtles (Chiari *et al*., 2012; Crawford *et al*., 2012, 2015; Fong *et al*., 2012). This accords with our earlier observation on the lack of tonus waves in atrial strips from crocodilians, lizards, or snakes (Galli *et al*., 2006; Joyce *et al*., 2014).

The atrial smooth muscle was particularly extensive in *T. scripta*, a species in the family (Emydidae) in which it was first described and detailed (Fano, 1887; Bottazzi, 1906; Gruber and Markel, 1918a; Shaner, 1923). Although we only studied one Emydid species in our phylogenetic survey, the extensive functional data suggest that atrial smooth muscle is well developed across this family as tonus waves are prevalent in atrial preparations species in both major subfamilies (Emydinae, e.g., *E. orbicularis*; Fano, 1887) (Deirochelyinae, e.g., *T. scripta*; Joyce *et al*., 2014). Even in *T. scripta*, smooth muscle was sparse in the ventricle, which concords with Shaner's (1923) anatomical description, and the fact that Fano only observed ventricular tonus thrice in over 100 experiments (Fano, 1887). It is surprising that tonus waves have been reported in *C. serpentina* (Pereira, 1924), including in the ventricle (Blinks and Koch‐Weser, 1963), given its sparse distribution, although the earlier descriptions in this species were vague. Our anatomical data consolidate the absence of atrial tonus in land tortoises (Testudinidae) (Gaskell, 1900).

### Smooth Muscle in the Hearts of Other Vertebrates

The phylogenetic distribution of atrial smooth muscle within the hearts of nonreptilian vertebrates remains somewhat enigmatic. Bottazzi (1897)) provided a description of atrial tonus in anuran amphibians (*Bufo viridis* and *Pelophylax esculentus*), but these findings received little subsequent attention and were not independently verified (Blinks and Koch‐Weser, 1963). Although we were able to see smooth muscle in the sinus venosus of both *X. laevis* and *R. marinus*, we did not locate smooth muscle in the atria. This is consistent with previous functional studies on isolated atrial preparations in these two species which did not report on tonus waves, even upon treatment with adenosine triphosphate (ATP) or acetylcholine (O'Donnell and Wanstall, 1982; Meghji and Burnstock, 1983; Minerds and Donald, 1997), which certainly activates the smooth muscle tones in atrial preparations from turtles (Fano, 1887; Joyce *et al*., 2014). Furthermore, although SMA is expressed transiently in the embryonic heart of *X. laevis*, no expression was found in the adult *X. laevis* heart (Saint‐Jeannet *et al*., 1992; Warkman *et al*., 2005; Barillot *et al*., 2008).

We did, nevertheless, verify that human atria contain traces of smooth muscle (Nagayo, 1909; Douglas *et al*., 2006; Park *et al*., 2013). We cannot speculate on its possible function, but human atrium does not appear to exhibit tonus waves (Meyer *et al*., 1996; Maier *et al*., 2000), including after exposure to acetylcholine (Nadler *et al*., 2011), thus we do not necessarily suggest it is functionally equivalent to atrial smooth muscle in turtles. It nevertheless demonstrates that atrial smooth muscle, *per se*, may not be unique to the turtle lineage.

### Pulmonary Veins of Turtles

Extensions of left atrial myocardium that partially envelop the pulmonary veins, known as “myocardial sleeves,” have been well described in mammals and birds (Nathan and Gloobe, 1970; Roux *et al*., 2004; Kroneman *et al*., 2019), but were not seen in corn snakes or anole lizards (Jensen *et al*., 2013). Where we could clearly observe the pulmonary veins in *T. scripta* (Fig. 6B), it appeared that there may be a small quantity of myocardium surrounding the vein, but this is not well developed.

## CONCLUSIONS

In summary, our comparative study indicates that atrial smooth muscle evolved early in the order of Testudines. The atrial smooth muscle is particularly scarce in terrestrial tortoises, but well developed in some aquatic species, which lends tentative support to our hypothesis that it may be involved in the regulation of cardiac output of turtles during diving. All of the turtles investigated exhibited both smooth muscle and cardiac muscle in the sinus venosus, which may also be able to contribute to the regulation of cardiac output. A mixture of smooth and cardiac muscle in the sinus venosus was also evident in the anuran amphibians and has previously been reported in fish (Yamauchi, 1980; Icardo, 2017), so we believe it likely represents the ancestral state in vertebrates. Although human atrial tissue also contains traces of smooth muscle, there is little indication that it is functionally homologous to that in turtles.

## References

[ar24257-bib-0001] Barillot W , Tréguer K , Faucheux C , Fédou S , Thézé N , Thiébaud P . 2008 Induction and modulation of smooth muscle differentiation in Xenopus embryonic cells. Dev Dyn 237:3373–3386. 10.1002/dvdy.21749. 18855898

[ar24257-bib-0103] Blix AS. 2018 Adaptations to deep andprolonged diving in phocid seals. J Exp Biol 221:jeb182972. 10.1242/jeb.182972 29934417

[ar24257-bib-0002] Blinks JR , Koch‐Weser J . 1963 Physical factors in the analysis of the actions of drugs on myocardial contractility. Pharmacol Rev 15:531–599. 14064357

[ar24257-bib-0003] Bottazzi P . 1897 The oscillations of the auricular tonus in the batrachian heart, with a theory on the function of sarcoplasma in muscular tissues. J Physiol 21:1–21. 10.1113/jphysiol.1897.sp000639. PMC1512985 16992370

[ar24257-bib-0004] Bottazzi P . 1906 Ricerche sulla muscolatura cardiale dell *Emys europaea* . Z Allg Physiol 6: 140.

[ar24257-bib-0005] Bottazzi P , Grünbaum OFF . 1899 On plain muscle. J Physiol 24:51–71. 10.1113/jphysiol.1899.sp000750. PMC1516622 16992482

[ar24257-bib-0006] Carmona R , Ariza L , Cañete A , Muñoz‐Chápuli R . 2018 Comparative developmental biology of the cardiac inflow tract. J Mol Cell Cardiol 116:155–164. 10.1016/j.yjmcc.2018.02.004. 29452155

[ar24257-bib-0007] Chiari Y , Cahais V , Galtier N , Delsuc F . 2012 Phylogenomic analyses support the position of turtles as the sister group of birds and crocodiles (Archosauria). BMC Biol 10:65 10.1186/1741-7007-10-65. PMC3473239 22839781

[ar24257-bib-0008] Conklin DJ , Lillywhite HB , Bishop B , Hargens AR , Olson KR . 2009 Rhythmic contractility in the hepatic portal “corkscrew” vein of the rat snake. Comp Biochem Physiol A Mol Integr Physiol 152:389–397. 10.1016/j.cbpa.2008.11.013. 19049826

[ar24257-bib-0009] Crawford NG , Faircloth BC , McCormack JE , Brumfield RT , Winker K , Glenn TC . 2012 More than 1000 ultraconserved elements provide evidence that turtles are the sister group of archosaurs. Biol Lett 8:783–786. 10.1098/rsbl.2012.0331. 22593086 PMC3440978

[ar24257-bib-0010] Crawford NG , Parham JF , Sellas AB , Faircloth BC , Glenn TC , Papenfuss TJ , Henderson JB , Hansen MH , Simison WB . 2015 A phylogenomic analysis of turtles. Mol Phylogenet Evol 83:250–257. 10.1016/j.ympev.2014.10.021. 25450099

[ar24257-bib-0011] de Bakker DM , Wilkinson M , Jensen B . 2015 Extreme variation in the atrial septation of caecilians (Amphibia: Gymnophiona). J Anat 226:1–12. 10.1111/joa.12255. 25400089 PMC4313894

[ar24257-bib-0012] Dimond MT . 1959 Responses to phenethylamines and nicotine and histology of turtle atria. Am J Physiol 197:747–751. 10.1152/ajplegacy.1959.197.4.747. 13816823

[ar24257-bib-0013] Douglas YL , Jongbloed MR , Gittenberger‐de Groot AC , Evers D , Dion RA , Voigt P , Bartelings MM , Schalij MJ , Ebels T , DeRuiter MC . 2006 Histology of vascular myocardial wall of left atrial body after pulmonary venous incorporation. Am J Cardiol 97:662–670. 16490434 10.1016/j.amjcard.2005.11.019

[ar24257-bib-0014] Douglas YL , Mahtab EA , Jongbloed MR , Uhrin P , Zaujec J , Binder BR , Schalij MJ , Poelmann RE , Deruiter MC , Gittenberger‐de Groot AC . 2009 Pulmonary vein, dorsal atrial wall and atrial septum abnormalities in podoplanin knockout mice with disturbed posterior heart field contribution. Pediatr Res 65:27–32. 18784615 10.1203/PDR.0b013e31818bc11a

[ar24257-bib-0100] Elsner R , Hanafee WN , Hammond DD . 1971 Angiography of the inferior vena cava of the harbor seal duringsimulated diving. Am J Physiol 220:1155–1157. 5574630 10.1152/ajplegacy.1971.220.5.1155

[ar24257-bib-0015] Fano G . 1887 Ueber die Tonusschwankungen der Atrien des Herzens von Emys europaea. Leipzig: JB Hirschfeld p 287–301.

[ar24257-bib-0016] Fano G , Bodano F . 1900 Sur les causes et sur la signification des oscillations du tonus auriculaire dans le coeur de l'*Emys europaea* . Arch Ital Biol 34:301–340.

[ar24257-bib-0017] Fano G , Fayod V . 1888 De quelques rapports entre les propriétés contractiles et les propriétés electriques des oreillettes du coeur. Arch Ital de Biol 4:61.

[ar24257-bib-0019] Fong JJ , Brown JM , Fujita MK , Boussau B . 2012 A phylogenomic approach to vertebrate phylogeny supports a turtle‐archosaur affinity and a possible paraphyletic Lissamphibia. PLOS One 7:e48990 10.1371/journal.pone.0048990. 23145043 PMC3492174

[ar24257-bib-0020] Galli GLJ , Gesser H , Taylor EW , Shiels HA , Wang T . 2006 The role of the sarcoplasmic reticulum in the generation of high heart rates and blood pressures in reptiles. J Exp Biol 209:1956–1963. 10.1242/jeb.02228. 16651560

[ar24257-bib-0021] Gannon BJ , Campbell GD , Thomas AC , Snyder GK . 2003 Endocardial smooth muscle in an Australian and two North American tortoises: cardiac tonus waves revisited 75 years on. Comp Biochem Physiol 134A:S112.

[ar24257-bib-0022] Gaskell WH . 1900 The contraction of cardiac muscle In: Schæfer EA, editor. Textbook of physiology. Edinburgh and London: Young J. Pentland p 169–227.

[ar24257-bib-0023] Gault CC . 1917 The physiology of the atrio‐ventricular connection in the turtle. Am J Physiol Leg Cont 43:22–42. 10.1152/ajplegacy.1917.43.1.22.

[ar24257-bib-0024] Glass M , Burggren WW , Johansen K . 1978 Ventilation in an aquatic and a terrestrial chelonian reptile. J Exp Biol 72:165–179. 624895 10.1242/jeb.72.1.165

[ar24257-bib-0025] Gruber CM . 1920a Iii. A note on the action of pilocarpine, atropine and adrenaline upon the tonus waves in the terrapin heart. J Pharmacol Exp Ther 15:23–28.

[ar24257-bib-0026] Gruber CM . 1920b Iv. The antagonistic actions of epinephrin and potassium chloride on the tonus and tonus waves in the excised terrapin auricles. J Pharmacol Exp Ther 15:271–277.

[ar24257-bib-0027] Gruber CM . 1921 V. Further studies on the antagonistic action of epinephrin to certain drugs upon the tonus and tonus waves in the terrapin auricles. J Pharmacol Exp Ther 16:405–413.

[ar24257-bib-0028] Gruber CM . 1927 Vi. Further studies on the effect of drugs upon the tonus waves in the excised terrapin auricles. J Pharmacol Exp Ther 31:333–352.

[ar24257-bib-0029] Gruber CM . 1934 A note on the effect of epinephrine on the excised terrapin sino‐auricular and atjricular apex strips. J Pharmacol Exp Ther 52:23–29.

[ar24257-bib-0030] Gruber CM , Markel C . 1918a I. Tonus waves from the sino‐auricular muscle preparation of the terrapin as affected by adrenalin. J Pharmacol Exp Ther 12:43–51.

[ar24257-bib-0031] Gruber CM , Markel C . 1918b Ii. Tonus waves in the terrapin auricles as affected by pilocarpine, atropine, and adrenalin. J Pharmacol Exp Ther 12:53–57.

[ar24257-bib-0101] Harrison RJ , Tomlinson JDW . 1956 Observations on the Venous System in Certain Pinnipedia and Cetacea. Proceedings of the Zoological Society of London 126:205–234.

[ar24257-bib-0032] Icardo JM . 2017 1—Heart morphology and anatomy In: Gamperl AK, Gillis TE, Farrell AP, Brauner CJ, editors. Fish physiology: The cardiovascular system. Cambridge, MA: Academic Press p 1–54. 10.1016/bs.fp.2017.05.002.

[ar24257-bib-0033] Jensen B , Boukens BJD , Wang T , Moorman AFM , Christoffels VM . 2014 Evolution of the sinus venosus from fish to human. J Cardiovasc Dev Dis 1:14–28. 10.3390/jcdd1010014.

[ar24257-bib-0034] Jensen B , Elfwing M , Elsey RM , Wang T , Crossley DA . 2016 Coronary blood flow in the anesthetized American alligator (*Alligator mississippiensis*). Comp Biochem Physiol Part A Mol Integr Physiol 191:44–52. 10.1016/j.cbpa.2015.09.018. 26436857

[ar24257-bib-0035] Jensen B , van den Berg G , van den Doel R , Oostra R‐J , Wang T , Moorman AFM . 2013 Development of the hearts of lizards and snakes and perspectives to cardiac evolution. PLOS One 8:e63651 10.1371/journal.pone.0063651. 23755108 PMC3673951

[ar24257-bib-0036] Jensen B , Vesterskov S , Boukens BJ , Nielsen JM , Moorman AFM , Christoffels VM , Wang T . 2017 Morpho‐functional characterization of the systemic venous pole of the reptile heart. Sci Rep 7 10.1038/s41598-017-06291-z. PMC5532247 28751678

[ar24257-bib-0037] Joyce W , Axelsson M , Wang T . 2019 Contraction of atrial smooth muscle reduces cardiac output in perfused turtle hearts. J Exp Biol. 222:jeb199828 10.1242/jeb.199828. 30787139

[ar24257-bib-0039] Joyce W , Gesser H , Wang T . 2014 Purinoceptors exert negative inotropic effects on the heart in all major groups of reptiles. Comp Biochem Physiol Part A Mol Integr Physiol 171:16–22. 10.1016/j.cbpa.2014.02.005. 24521885

[ar24257-bib-0040] Joyce W , Williams CJA , Crossley DA , Wang T . 2018 Venous pressures and cardiac filling in turtles during apnoea and intermittent ventilation. J Comp Physiol B Biochem Syst Environ Physiol 188:481–490. 10.1007/s00360-017-1132-3. 29071420

[ar24257-bib-0041] Kroneman JGH , Faber JW , Schouten JCM , Wolschrijn CF , Christoffels VM , Jensen B . 2019 Comparative analysis of avian hearts provides little evidence for variation among species with acquired endothermy. J Morphol. 280: 395–410. 10.1002/jmor.20952. 30667083 PMC6590421

[ar24257-bib-0042] Laurens H . 1913 The atrio‐ventricular connection in the reptiles. Anat Rec 7:273–285. 10.1002/ar.1090070803.

[ar24257-bib-0102] Lillie MA , Vogl AW , Raverty S , Haulena M , McLellan WA , Stenson, GB , Shadwick, RE. 2018 The cavalsphincter in cetaceans and its predicted role in controlling venous flow duringa dive. J Exp Biol 221:jeb.177212 10.1242/jeb.177212 29674378

[ar24257-bib-0043] Lillywhite HB . 1987 Circulatory adaptations of snakes to gravity. Integr Comp Biol 27:81–95. 10.1093/icb/27.1.81.

[ar24257-bib-0044] Maier LS , Barckhausen P , Weisser J , Aleksic I , Baryalei M , Pieske B . 2000 Ca^2+^ handling in isolated human atrial myocardium. Am J Physiol Heart Circulat Physiol 279:H952–H958. 10.1152/ajpheart.2000.279.3.H952. 10993755

[ar24257-bib-0045] Meghji P , Burnstock G . 1983 An unusual excitatory action of adenosine on the ventricular muscle of the South African clawed toad (*Xenopus laevis*). Eur J Pharmacol 89:251–258. 6873160 10.1016/0014-2999(83)90501-0

[ar24257-bib-0046] Meyer M , Lehnart S , Pieske B , Schlottauer K , Munk S , Holubarsch C , Just H , Hasenfuss G . 1996 Influence of endothelin 1 on human atrial myocardium–myocardial function and subcellular pathways. Basic Res Cardiol 91:86–93. 8660266 10.1007/BF00788869

[ar24257-bib-0047] Minerds KL , Donald JA . 1997 Lack of evidence for functional natriuretic peptide receptors in the heart of the cane toad, *Bufo marinus* . Comp Biochem Physiol C Pharmacol Toxicol Endocrinol 118:233–240. 9440250 10.1016/s0742-8413(97)00134-5

[ar24257-bib-0048] Moretti A , Caron L , Nakano A , Lam JT , Bernshausen A , Chen Y , Qyang Y , Bu L , Sasaki M , Martin‐Puig S , et al. 2006 Multipotent embryonic isl1+ progenitor cells lead to cardiac, smooth muscle, and endothelial cell diversification. Cell 127:1151–1165. 17123592 10.1016/j.cell.2006.10.029

[ar24257-bib-0049] Nadler E , Barnea O , Vidne B , Isakov A , Shavit G . 2011 Positive inotropic effect in the heart produced by acetylcholine. J Basic Clin Physiol Pharmacol 4:229–248. 10.1515/JBCPP.1993.4.3.229. 8679518

[ar24257-bib-0050] Nagayo M . 1909 Zur normalen und pathologischen Histologie des Endocardium parietale. Beitr Path Anat 45:283–305.

[ar24257-bib-0051] Nathan H , Gloobe H . 1970 Myocardial atrio‐venous junctions and extensions (sleeves) over the pulmonary and caval veins. Anatomical observations in various mammals. Thorax 25:317–324. 5452285 10.1136/thx.25.3.317 PMC472709

[ar24257-bib-0052] O'Donnell SR , Wanstall JC . 1982 Pharmacological experiments demonstrate that toad (*Bufo marinus*) atrial beta‐adrenoceptors are not identical with mammalian beta 2‐ or beta 1‐adrenoceptors. Life Sci 31:701–708. 6127586 10.1016/0024-3205(82)90772-x

[ar24257-bib-0053] Oinuma S . 1910 Beiträge zur Physiologie der autonom innervierten Muskulatur. Pflüger's Arch 133:500–518. 10.1007/BF01705453.

[ar24257-bib-0054] Okada H , Takemura G , Kanamori H , Tsujimoto A , Goto K , Kawamura I , Watanabe T , Morishita K , Miyazaki N , Tanaka T , et al. 2015 Phenotype and physiological significance of the endocardial smooth muscle cells in human failing hearts. Circ Heart Fail 8:149–155. 10.1161/CIRCHEARTFAILURE.114.001746. 25466765

[ar24257-bib-0055] Park JH , Pak H‐N , Lee S , Park HK , Seo J‐W , Chang B‐C . 2013 The clinical significance of the atrial subendocardial smooth muscle layer and cardiac myofibroblasts in human atrial tissue with valvular atrial fibrillation. Cardiovasc Pathol 22:58–64. 10.1016/j.carpath.2012.05.001. 22658273

[ar24257-bib-0056] Pereira JR . 1924 On the influence of temperature on the tonus waves of the turtle auricle. Am J Physiol Leg Cont 70:68–72. 10.1152/ajplegacy.1924.70.1.68.

[ar24257-bib-0057] Robb JS . 1952 Specialized (conducting) tissue in the turtle heart. Am J Physiol Leg Cont 172:7–13. 10.1152/ajplegacy.1952.172.1.7. 13030707

[ar24257-bib-0058] Rosenzweig E . 1903 Beiträge zur Kenntniss der Tonus Schwankungen des Herzens van *Emys europaea* . Arch Anat Physiol 192: 92.

[ar24257-bib-0059] Roux N , Havet E , Mertl P . 2004 The myocardial sleeves of the pulmonary veins: potential implications for atrial fibrillation. Surg Radiol Anat 26:285–289. 10.1007/s00276-003-0219-6. 14872285

[ar24257-bib-0060] Saint‐Jeannet JP , Levi G , Girault JM , Koteliansky V , Thiery JP . 1992 Ventrolateral regionalization of *Xenopus laevis* mesoderm is characterized by the expression of alpha‐smooth muscle actin. Development 115:1165–1173. 1451663 10.1242/dev.115.4.1165

[ar24257-bib-0061] Shaffer HB , McCartney‐Melstad E , Near TJ , Mount GG , Spinks PQ . 2017 Phylogenomic analyses of 539 highly informative loci dates a fully resolved time tree for the major clades of living turtles (Testudines). Mol Phylogenet Evol 115:7–15. 10.1016/j.ympev.2017.07.006. 28711671

[ar24257-bib-0062] Shaner RF . 1923 On the smooth muscle in the turtle's heart. Anat Rec 25:71–75. 10.1002/ar.1090250203.

[ar24257-bib-0063] Sollmann T , Rossides TN . 1927 The effects of epinephrine on the auricular tonus waves of the turtle heart. J Pharmacol Exp Ther 32:19–22.

[ar24257-bib-0064] Taylor EW , Wang T . 2009 Control of the heart and of cardiorespiratory interactions in ectothermic vertebrates In: Glass ML, Wood SC, editors. Cardio‐respiratory control in vertebrates. Berlin, Heidelberg: Springer Verlag p 285–315.

[ar24257-bib-0065] Terasaki F , James TN , Hayashi T . 1993 Electron microscopic demonstration of intercellular junctions between subendocardial smooth muscle and myocardium in the sheep heart. Am Heart J 126:399–405. 8338011 10.1016/0002-8703(93)91057-l

[ar24257-bib-0066] Wang T , Hicks JW . 1996 Cardiorespiratory synchrony in turtles. J Exp Biol 199:1791–1800. 8708580 10.1242/jeb.199.8.1791

[ar24257-bib-0067] Warkman AS , Zheng L , Qadir MA , Atkinson BG . 2005 Organization and developmental expression of an amphibian vascular smooth muscle alpha‐actin gene. Dev Dyn 233:1546–1553. 10.1002/dvdy.20457. 15965984

[ar24257-bib-0068] Yamauchi A . 1980 Fine structure of the fish heart In: Bourne G, editor. Heart and heart‐like organs. New York: Academic Press p 119–148.

